# Efficacy and safety of Buyang Huanwu Decoction in the treatment of post-stroke depression: A systematic review and meta-analysis of 15 randomized controlled trials

**DOI:** 10.3389/fneur.2022.981476

**Published:** 2022-11-04

**Authors:** Kun Zhen, Hongshuo Shi, Xuecheng Zhang, Xiangyi Liu, Wenwen Li, Guomin Si, Hongling Jia, Dong Guo

**Affiliations:** ^1^College of Traditional Chinese Medicine, Shandong University of Traditional Chinese Medicine, Jinan, China; ^2^Key Laboratory of Chinese Internal Medicine of Ministry of Education, Dongzhimen Hospital, Beijing University of Chinese Medicine, Beijing, China; ^3^The First Clinical Medical College, Beijing University of Chinese Medicine, Beijing, China; ^4^Department of Traditional Chinese Medicine, Provincial Hospital Affiliated to Shandong First Medical University, Jinan, China; ^5^The Second Affiliated Hospital of Shandong University of Traditional Chinese Medicine, Jinan, China; ^6^The First Affiliated Hospital of Shandong University of Traditional Chinese Medicine, Jinan, China

**Keywords:** Buyang Huanwu Decoction, post-stroke depression, systematic reviews, meta-analyses, overview

## Abstract

**Background:**

Post-stroke depression is the most common neuropsychiatric disorder after stroke, which seriously affects patients' post-stroke recovery and quality of life, and is prone to recurrence of stroke and death. Buyang Huanwu Decoction is effective in treating post-stroke depression, but there is a lack of scientific systematic review and meta-analysis.

**Objective:**

To evaluate the efficacy and safety of Buyang Huanwu Decoction in treating post-stroke depression.

**Methods:**

A total of eight databases were searched by two investigators from Embase, PubMed, The Cochrane Library, Web of Science, Wanfang, CNKI, VIP, and CBM to collect randomized controlled trials that applied BHD to PSD from the time of database construction to May 2022. Data analysis was performed using Review mange5.4.

**Results:**

A total of 15 studies with 1,242 patients were included. Meta-analysis showed that compared with the antidepressant drug control group, the change value of the HAMD scale in the Buyang Huanwu Decoction group was significantly lower [*p* < 0.00001, SMD = −0.85, 95% CI (−1.10, −0.61)]; after subgroup analysis, the effect of BHD for 4 weeks was the most significant; the total clinical effective rate was significantly increased [*p* = 0.001, RR = 1.33, 95% CI (1.12, 1.57)]; neurological deficit score [*p* = 0.002, SMD = −1.03, 95% CI (−1.67, −0.39)], the incidence of adverse reactions [*p* = 0.02, RR = 0.42, 95% CI (0.20, 0.89)], and adverse reaction scale scores [*p* < 0.00001, MD = −3.58, 95%CI (−4.09, −3.08)] were significantly lower.

**Conclusion:**

Compared with antidepressants, the Buyang Huanwu Decoction is more effective and safer in the treatment of post-stroke depression patients. However, more high-quality studies are needed to further support the above conclusion.

## Introduction

Stroke is one of the leading causes of death and disability in the world, although the mortality and burden of stroke vary considerably across countries (1). Among them, post-stroke depression (PSD) is the most common stroke neuropsychiatric sequelae, and the prevalence of PSD is 11–41% since 2019 (2), and the cumulative incidence of depression was 52% 5 years after stroke (3). PSD is the main factor of poor recovery, poor quality of life, and poor rehabilitation after stroke (4). At the same time, the probability of stroke recurrence and death in PSD patients is higher than that in non-PSD patients (5, 6). In contrast, early treatment of PSD can enhance the recovery of physical function and cognition after stroke, and increase the survival rate of patients 10 years after stroke (7).

Currently, PSD is severely under-diagnosed and under-treated and is best treated with a combination of pharmacological, psychosocial, and stroke-focused treatment, which includes restoration of limb function and psycho-cognition, and prevention of new acute vascular events (8). A wide range of antidepressants is commonly used in clinical practice, such as selective serotonin reuptake inhibitors (SSRIs), tricyclic antidepressants (TCAs), and serotonin-norepinephrine reuptake inhibitors (SNRIs), which allow depressive symptoms to be significantly improved (9–11). Studies have shown that the use of SSRIs in the early stages of stroke not only reduces the incidence of PSD but also promotes neuronal regeneration and plasticity changes, which facilitate the recovery of nerve function, cognitive and language functions, and improve the prognosis of patients after stroke (12, 13). However, compared with a placebo, SSRIs were only significantly effective for mild depression and did not improve motor and cognitive function or quality of life in patients with PSD, nor did they significantly improve moderate to severe depression (14). Simultaneously, long-term use of antidepressants is likely to cause gastrointestinal dysfunction and increase the risk of cerebrovascular events (15).

In recent years, Chinese herbal medicine as a complementary or alternative therapy has been introduced to treat PSD, and studies have reported excellent antidepressant efficacy and a reduced incidence of adverse effects (16–19). Buyang Huanwu Decoction (BHD) is a classic Chinese medicine prescription for treating stroke and its sequelae. Experimental studies have confirmed that BHD can improve nerve injury, reduce nerve apoptosis, reduce oxidative stress injury, induce cell regeneration, and so on (20–23). At present, BHD alone or in combination with conventional antidepressants has been widely used in the treatment of post-stroke depression in China (24–38). However, there is still a lack of reliable systematic review and meta-analysis of BHD therapy for PSD. The purpose of this study is to comprehensively investigate the efficacy and safety of BHD in the treatment of PSD, which is as follows: (1) To determine whether BHD is effective compared with conventional antidepressants; and (2) to compare the incidence of adverse reactions of BHD and determine the safety of BHD in treating PSD.

## Data and methods

### Inclusion criteria

#### Research type

A randomized controlled trial of BHD in the treatment of PSD. The language of publication is limited to Chinese and English.

#### Research objects

Patients diagnosed with post-stroke depression are the research objects. The diagnosis of stroke is based on “Key Points for Diagnosing Cerebrovascular Diseases” adopted by the 4th China Academic Conference on Cerebrovascular Diseases, and the diagnosis is made by cranial CT or MRI. The diagnosis of post-stroke depression is based on the Chinese classification and diagnosis standard of the mental disorders 3rd edition (CCMD-3) or the Chinese classification scheme and diagnosis standard of mental disorders. The race, sex, age, onset time, and course of disease of patients are not limited.

#### Intervention

The control group received basic stroke treatment, mainly for controlling blood pressure, blood sugar, blood lipids, anti-platelet aggregation, nourishing brain nerves, improving cerebral vascular microcirculation, and preventing complications; or basic treatment plus conventional antidepressant drug treatment, including fluoxetine tablets, fluoxetine capsules, paroxetine hydrochloride tablets, flupentixol, melitracen tablets, and mirtazapine; or only conventional antidepressant drug treatment. The intervention group was given BHD, or added BHD based on the control group.

#### Outcomes

The main outcome measures included the changes in scores of 17, 21, and 24-item Hamilton Depression Scale (HAMD) before and after treatment. The secondary outcome measures were the total clinical response rate, neurological deficit score, theincidence of adverse reactions, and the Treatment Emergent Symptom Scale (TESS).

The effective rate was classified according to the HAMD score reduction rate before and after treatment. Recovery: the reduction rate is >90%, markedly effective: the reduction rate is 75–89%, effective: the reduction rate is 50–74%, and ineffective: the reduction rate is <50%. In addition, clinical efficacy is divided into effective (including recovery, markedly effective, and effective) and ineffective.

### Exclusion criteria

Basic research literature such as review, meta-analysis, and experience of famous doctors; non-clinical research literature such as animal experiments and mechanism interpretation; clinical research literature of non-randomized controlled trials; literature on intervention measures other than BHD; not using BHD Literature as primary treatment; literature with incomplete or erroneous data; literature with duplicate publications or incomplete outcome measures; academic dissertation.

### Retrieval strategy

Two researchers jointly searched eight Chinese and English databases, including Embase, PubMed, The Cochrane Library, Web of Science, Wanfang, China Biomedical Literature Service System (CBM), China National Knowledge Infrastructure (CNKI), and VIP. The search time was until May 2022. The language of publication is limited to Chinese and English. Search subject words and free words in combination. The keywords include Buyang Huanwu Decoction, Buyang Huanwu tang, Buyang Huanwu formula, post-stroke depression, depression after stroke, post-stroke depressive disorder, and depressive disorder after stroke. The database search strategy was shown in Table 1.

**Table 1:** Search strategy to identify RCTs in databases.

**Databases**	**Search strategy**	**Items**
PubMed	((((((((((depression[Title/Abstract]) OR (stroke[Title/Abstract])) OR (post stroke[Title/Abstract])) OR (after stroke[Title/Abstract])) OR (post stroke depression[Title/Abstract])) OR (post-stroke depression[Title/Abstract])) OR (PSD[Title/Abstract])) OR (depression after stroke[Title/Abstract])) OR (post stroke depressive disorder[Title/Abstract])) OR (depressive disorder after stroke[Title/Abstract])) AND ((((Buyang Huanwu[Title/Abstract]) OR (Buyang Huanwu tang[Title/Abstract])) OR (Buyang Huanwu formula[Title/Abstract])) OR (Buyang Huanwu decoction[Title/Abstract]))	60
Web of Science	(“post stroke depression” OR “PSD” OR “depression after stroke” OR “post stroke depressive disorder” OR “depressive disorder after stroke”)AND (“Buyang Huanwu” OR “Buyang Huanwu tang” OR “Buyang Huanwu formula” OR “Buyang Huanwu decoction”)	1
Embase	(“post stroke depression” OR “PSD” OR “depression after stroke” OR “post stroke depressive disorder” OR “depressive disorder after stroke”) AND (“buyang huanwu” OR “buyang huanwu tang” OR “buyang huanwu formula” OR “buyang huanwu decoction”)	1
The Cochrane library	(“post stroke depression” OR “PSD” OR “depression after stroke” OR “post stroke depressive disorder” OR “depressive disorder after stroke”) AND (“buyang huanwu” OR “buyang huanwu tang” OR “buyang huanwu formula” OR “buyang huanwu decoction”)	0
Wanfang	((((((主题=补阳还五汤) OR 主题=补阳还五汤加减) OR 主题=补阳还五汤加味) OR 主题=补阳还五汤化裁))) AND (((((((((主题=中风后抑郁) OR 主题=中风后抑郁患者) OR 主题=中风后抑郁状态) OR 主题=中风后抑郁障碍) OR 主题=中风后抑郁症))) OR ((((((主题=卒中后抑郁) OR 主题=卒中后抑郁患者) OR 主题=卒中后抑郁状态) OR 主题=卒中后抑郁障碍) OR 主题=卒中后抑郁症(PSD)))))	39
CBM	(“补阳还五汤"[常用字段:智能]] OR “补阳还五汤加减"[常用字段:智能] OR “补阳还五汤加味"[常用字段:智能] AND ((“中风后抑郁"[常用字段:智能] OR “中风后抑郁症(PSD)”[常用字段:智能] OR “中风后抑郁状态"[常用字段:智能] OR “中风后抑郁障碍"[常用字段:智能] OR “中风后抑郁患者"[常用字段:智能]) OR (“卒中后抑郁"[常用字段:智能] OR “卒中后抑郁状态"[常用字段:智能] OR “卒中后抑郁障碍"[常用字段:智能] OR “卒中后抑郁患者"[常用字段:智能] OR “卒中后抑郁症(PSD)”[常用字段:智能]))	31
CNKI	(卒中后抑郁 + 卒中后抑郁症 + 卒中后抑郁患者 + 卒中后抑郁(psd)+ 卒中后抑郁状态 + 卒中后抑郁障碍)OR(中风后抑郁 + 中风后抑郁症 + 中风后抑郁(psd) + 中风后抑郁症患者 + 中风后抑郁证)AND (补阳还五汤+补阳还五汤加减+补阳还五汤加味)	42
VIP	(脑卒中后抑郁+depression after cerebral apoplexy+post-stroke depressive+poststroke depression+中风后抑郁+脑卒中后抑郁症+卒中后抑郁+卒中后抑郁症) OR (中风后抑郁+中风后抑郁障碍+中风后抑郁障碍+中风后抑郁状态) AND (补阳还五汤+补阳还五汤加减+补阳还五汤加味)	70

### Data extraction

Based on the inclusion and exclusion criteria, two researchers independently screened the literature, extracted data according to a pre-designed table, and cross-checked it.

To avoid disagreement, a third researcher was involved to help resolve it. Extracted content: title, name of the first author, year of publication, sample size, age and gender of the participants, intervention measures, treatment course, outcome indicators, and so on.

### Literature quality evaluation

Based on the seven criteria recommended by Cochrane Bias Risk Tool, two researchers independently evaluated the quality of RCTs. The evaluation contents included seven items: random sequence generation, allocation sequence concealment, blind method of researchers and subjects, blind method of outcome assessment, completeness of outcome data, selective reporting of research results, and other biases.

### Data analysis

Review Manager 5.4 software was used for statistical processing. Count data were described by relative risk (RR), while measurement data were described by mean difference (MD) or standardized mean difference (SMD). If there was good homogeneity between studies, a fixed-effects model was used for *P* ≥ 0.1 or *I*^2^ ≤ 50%; conversely, a random-effects model would be used. In case of significant heterogeneity, sensitivity analysis was performed as appropriate to assess the possible sources of heterogeneity emergence. Publication bias was assessed with funnel plots.

## Results

### Literature search and screening

**Figure 1:**
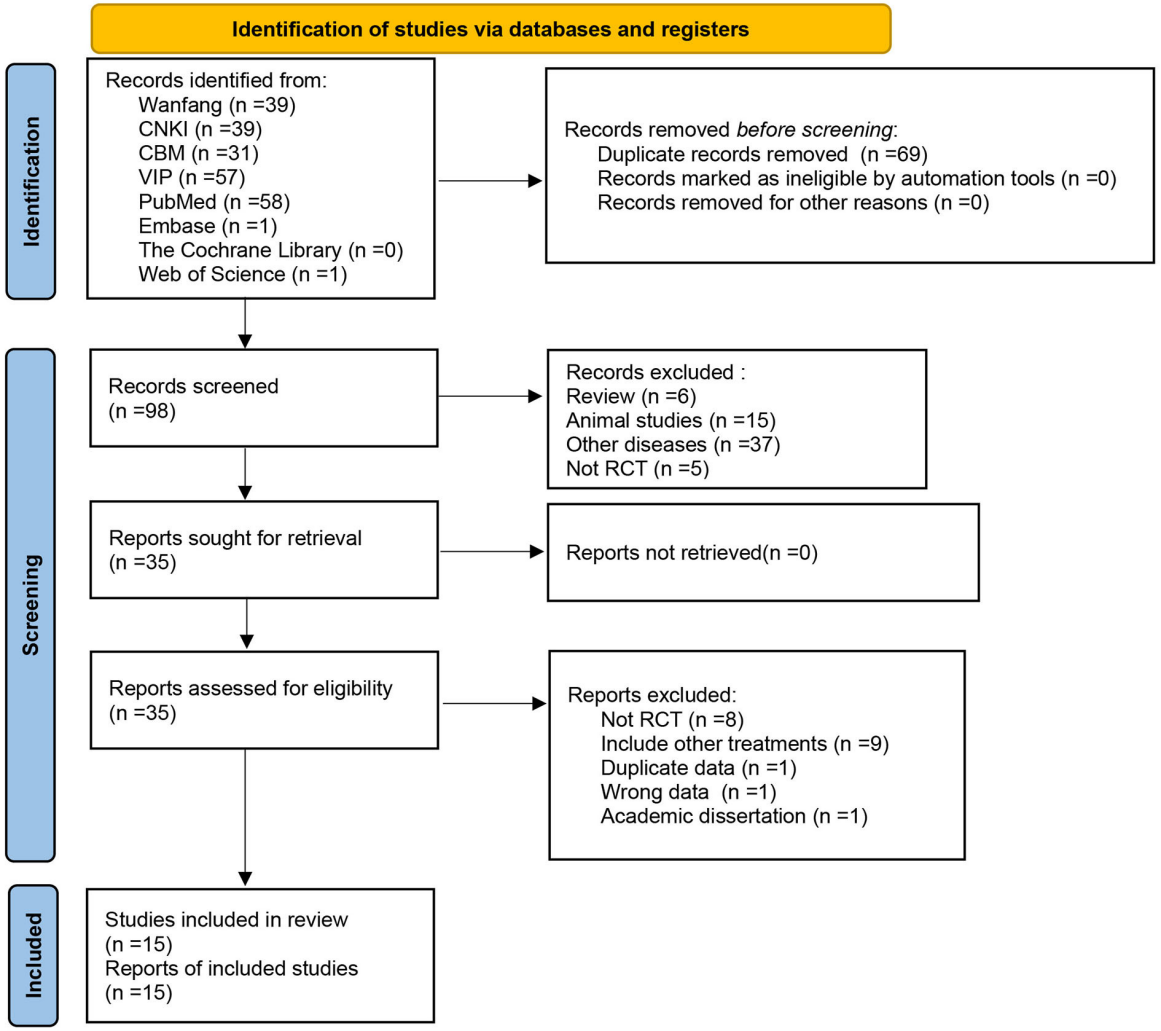
Flow diagram of literature screening.

Eight databases were initially checked for 167 relevant articles, and after checking by Endnote software, 69 duplicate articles were excluded, 35 articles were obtained after reading the article titles and abstracts, and 15 articles were finally included after further reading of the full text, all of which were in Chinese (Figure 1).

### Basic characteristics of the included literature

The 15 included RCT studies were all in Chinese and included a total of 1,242 patients, 623 patients in the intervention group, and 619 patients in the control group. The maximum sample size was 148 patients and the minimum sample size was 37 patients. Among them, 14 studies had clear diagnostic criteria. The average age of patients and the number of male and female cases were not reported in one study (24), one study (25) compared BHD with no drugs, six studies (24, 30–34) compared BHD with conventional antidepressant drugs, and eight studies (26–29, 35–38) compared the combination of BHD and antidepressant conventional drugs with antidepressant conventional drugs alone. Follow-ups ranged from 3 weeks to 3 months, with a mean of 6 weeks. The included studies all clearly stated that the intervention group was comparable to the control group at baseline. The basic information of the included studies is shown in Table 2.

**Table 2:** Basic information of the literature.

**Study**	**Number of participants**		**Age**		**Gender (male/female)**		**Baseline HAMD (mean** ± **SD)**		**Fund status**	**Dropouts**	**Comparisons**				
	**Experiment**	**Control**	**Experiment**	**Control**	**Experiment**	**Control**	**Experiment**	**Control**			**Experiment**	**Control**	**Diagnostic criteria**	**Depression degree**	**Duration (weeks)**
Zhou et al. (38)	41	40	62.3 ± 5.8	64.5 ± 6.2	25/16	23/17	23.62 ± 6.11	22.84 ± 5.13	Yes	No	BHD plus Fluoxetine	Fluoxetine	KPDCD, CCMD-3	18HAMD ≤ 35(24)	12 weeks
Sun (33)	26	24	66.55	65.23	15/11	13/11	21.35 ± 6.56	22.65 ± 6.76	NM	No	BHD	Fluoxetine	KPDCD,CT or MRI, CCMD-3	8 ≤ HAMD ≤ 35(NS)	4 weeks
Chen (24)	30	30	58–75		NM		NM		NM	No	BHD	Fluoxetine	NM	18 ≤ HAMD < 30(NS)	6 weeks
Xu et al. (34)	74	74	65.12 ± 7.51	63.58 ± 6.13	39/35	41/33	22.01 ± 7.12	21.93 ± 7.15	NM	No	BHD	Fluoxetine	CCMD-3	HAMD ≥ 7(NS)	12 weeks
Li (26)	45	45	53.7 ± 4.4	55.1 ± 5.4	25/20	22/23	25.55 ± 2.72	26.03 ± 2.94	NM	No	BHD plus Mirtazapine	Mirtazapine	KPDCD,CT or MRI, CCMD-3	18 ≤ HAMD ≤ 35(NS)	8 weeks
Nie et al. (30)	35	38	65.2 ± 9.25	62.1 ± 9.55	15/20	20/18	29.37 ± 4.33	28.48 ± 5.26	NM	7	BHD	Paroxetine Hydrochloride	KPDCD,CT or MRI, CCMD-3	HAMD ≥ 20(24)	4 weeks
Liu and Yang (28)	42	40	67.5 ± 6.7	66.7 ± 6.1	30/12	28/12	26.94 ± 4.70	27.14 ± 4.85	NM	No	BHD plus Paroxetine Hydrochloride	Paroxetine Hydrochloride	KPDCD,CT or MRI, CCMD-3	HAMD(NS)	4 weeks
Liu (29)	59	58	64.15 ± 6.15	63.99 ± 6.09	30/29	31/27	28.59 ± 8.75	28.61 ± 8.82	NM	No	BHD plus Flupentixol Melitracen Tablets	Flupentixol Melitracen Tablets	KPDCD	HAMD ≥ 8(NS)	3 weeks
Liao et al. (27)	22	22	64.27 ± 9.16	63.58 ± 8.42	14/8	12/10	22.47 ± 6.62	22.23 ± 6.83	NM	No	BHD plus Fluoxetine	Fluoxetine	KPDCD,CT or MRI, CCMD-3	HAMD ≥ 8(NS)	/
Xuan (35)	65	65	54.2 ± 6.5	54.7 ± 6.9	41/24	39/26	NM		NM	No	BHD plus Fluoxetine	Fluoxetine	NM	SDS(NS)	4 weeks
Ying (36)	35	35	62.12 ± 2.09	62.19 ± 2.00	21/14	19/16	24.96 ± 2.11	24.90 ± 2.18	NM	No	BHD plus Paroxetine Hydrochloride	Paroxetine Hydrochloride	CT or MRI	HAMD > 8(NS)	4 weeks
Su and Zhuang (32)	39	39	63.1 ± 10.5	63.8 ± 10.1	21/18	22/17	25.75 ± 4.33	26.02 ± 4.27	NM	No	BHD	Paroxetine Hydrochloride	CT or MRI, CCMD-3	HAMD ≥ 20(24)	4 weeks
Shao (31)	19	18	63.27 ± 2.05	63.28 ± 2.16	11/8	10/8	29.19 ± 2.30	28.96 ± 1.95	NM	No	BHD	Paroxetine Hydrochloride	CT or MRI, CCMD-3	HAMD(24)	4 weeks
Cheng et al. (25)	40	40	60.34 ± 4.52	60.47 ± 4.48	23/17	24/16	28.51 ± 2.76	28.49 ± 2.80	NM	No	BHD	no medicine	NM	HAMD(NS)	12 weeks
Zhao (37)	51	51	52.8 ± 2.6	52.2 ± 2.5	26/25	25/26	30.51 ± 4.82	30.19 ± 5.19	NM	No	BHD plus Mirtazapine	Mirtazapine	NM	HAMD(NS)	8 weeks

KPDCD, Key Points for Diagnosing Cerebrovascular Diseases; CCMD-3, Chinese Classification of Mental Disorders Version 3; HAMD, Hamilton depression scale; SDS, Self-rating depression scale; NM, not mentioned; NS, not stated the version of HAMD.

### Quality evaluation of the included studies

**Figure 2:**
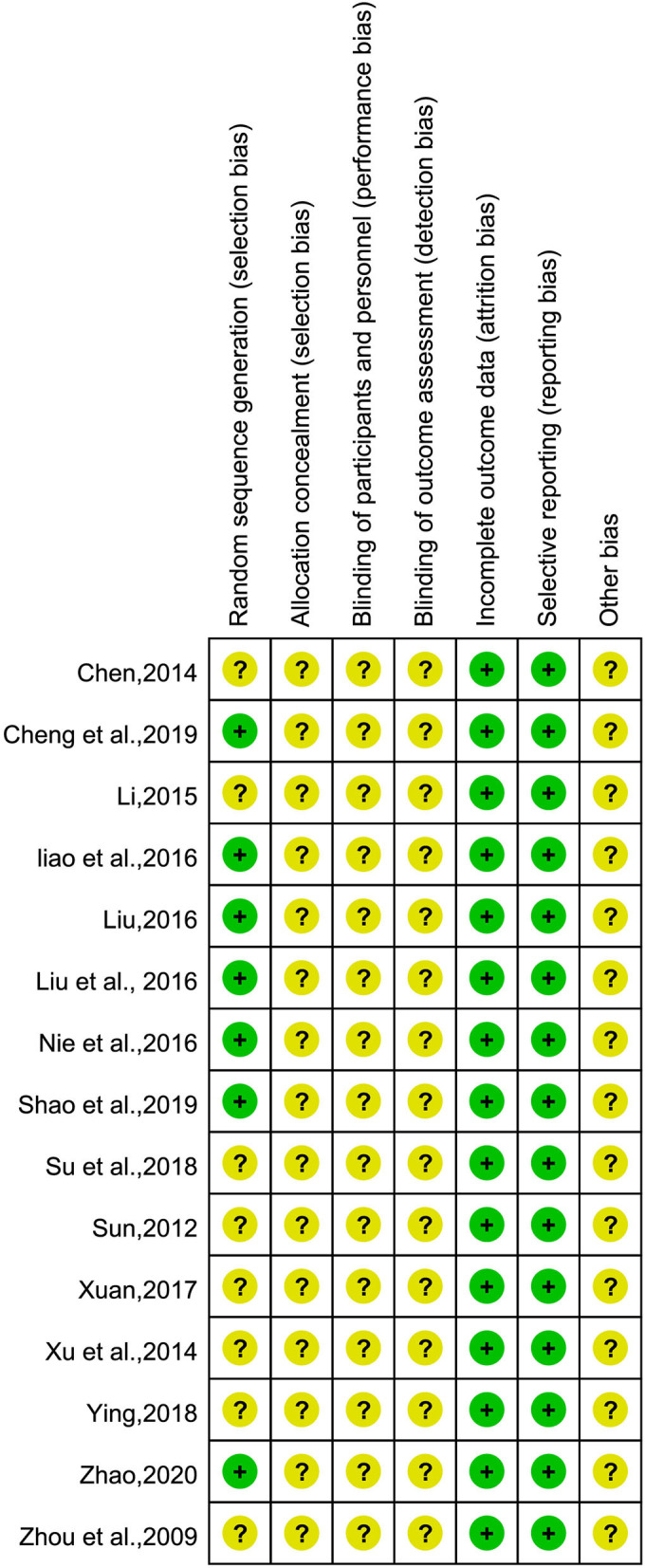
Summary of risk of bias.

The risks of bias assessment of the included studies are shown in Figure 2. The quality of the included studies was generally poor, in terms of random sequence generation, allocation concealment, and blinding, all of which did not provide sufficient information. Inadequate reporting will increase the risk of study bias. Seven studies (25, 27–31, 37) used the random number table method, while the remaining studies only mentioned the word “random”; 15 studies did not report the allocation concealment method or whether they were blinded; five studies (26, 27, 30, 36, 37) mentioned informed consent; and three studies (30, 36, 37) indicated that they passed the ethical review. One study (30) had case shedding and reported the data completely. It is unclear whether there are other biases in the included studies.

### Meta-analysis

#### The changes of HAMD

**Figure 3:**
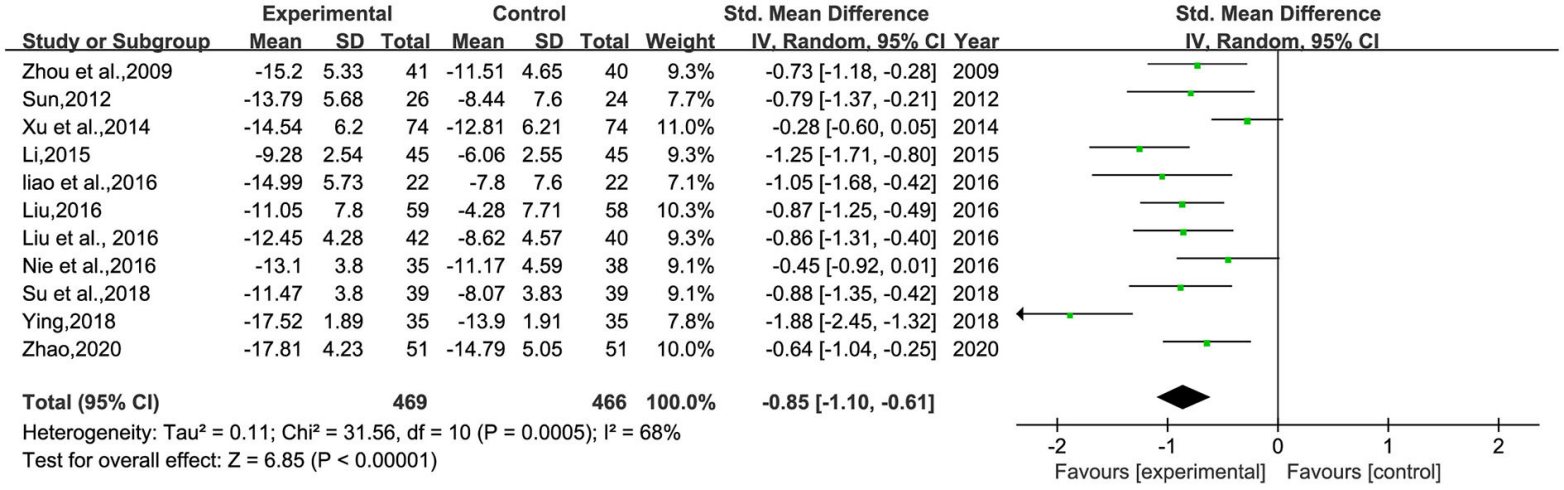
Meta-analysis of 1-session BHD and HAMD change values of antidepressant drugs. Green: Continuous.

**Figure 4:**
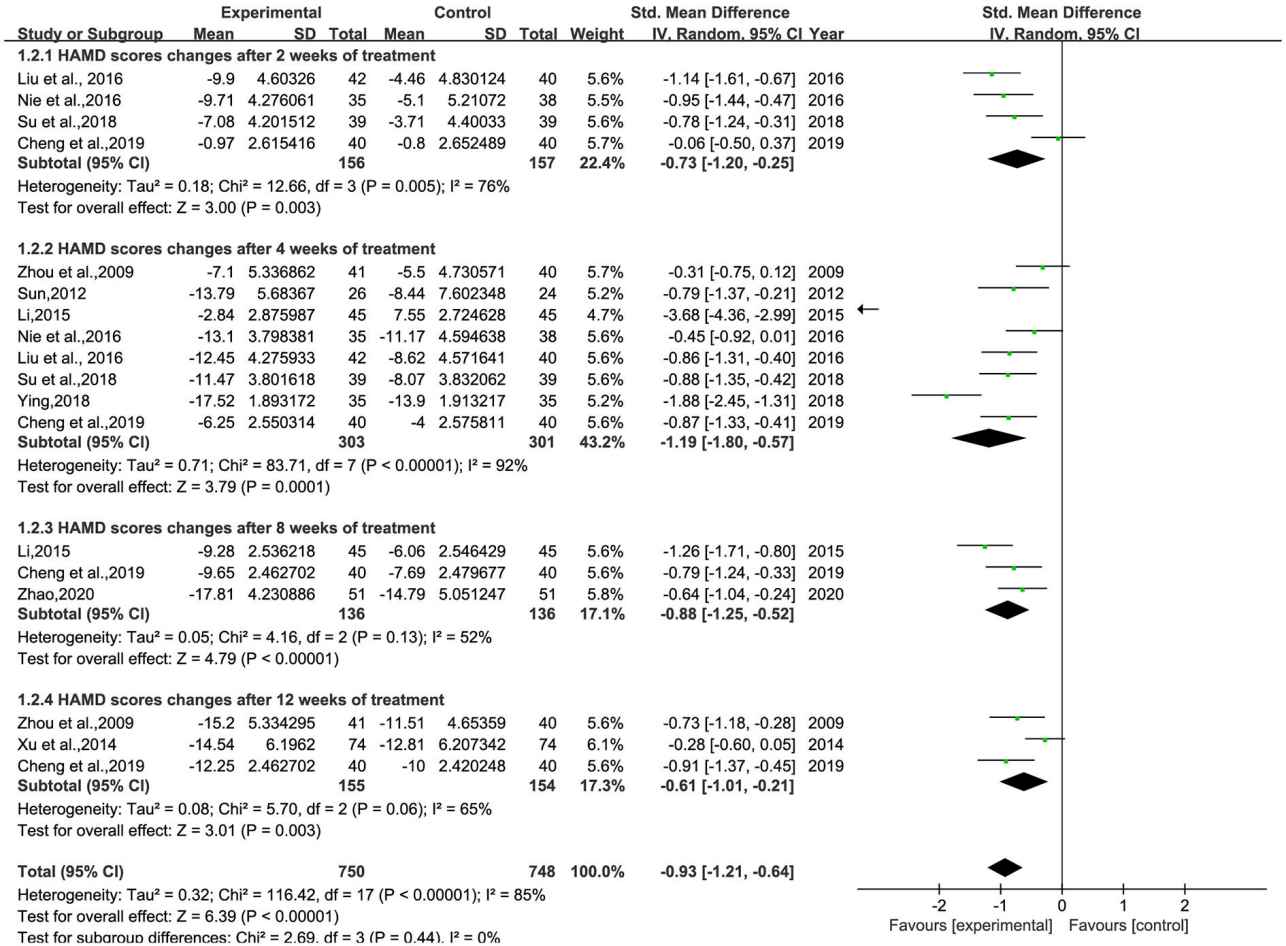
Meta-analysis of BHD and antidepressant medication cycles on HAMD change values. Green: Continuous.

A total of 11 studies (26–30, 32–34, 36–38) involving 935 patients were included (Figure 3). Heterogeneity was tested (*p* < 0.00001, *I*^2^ = 68%), and a meta-analysis was performed using a random-effects model. The results of the analysis showed that the intervention group was more effective in reducing HAMD scores than the control group. The difference was statistically significant [SMD = −0.85, 95% CI (−1.10, −0.61)]. Since the doses and frequencies of herbal medicines were consistent in the study, only the dosing cycles were analyzed in subgroups (Figure 4). The results of the analysis showed 2 weeks of medication [SMD = −0.73, 95% CI (−1.20, −0.25), *p* = 0.003], 4 weeks [SMD = −1.19, 95% CI (−1.80, −0.57), *p* = 0.0001], 8 weeks [SMD = −0.88, 95% CI (−1.25, −0.52), *p* < 0.00001], 12 weeks [SMD = −0.61, 95% CI (−1.01, −0.21), *p* = 0.003], and overall results [SMD = −0.93, 95% CI (−1.21, −0.64), *p* < 0.00001]. The analysis showed that the change of HAMD was the most obvious after 4 weeks of BHD administration. The reason of heterogeneity may be related to the different types of antidepressants and the standard of the HAMD scale.

#### Total clinical effective rate

**Figure 5:**
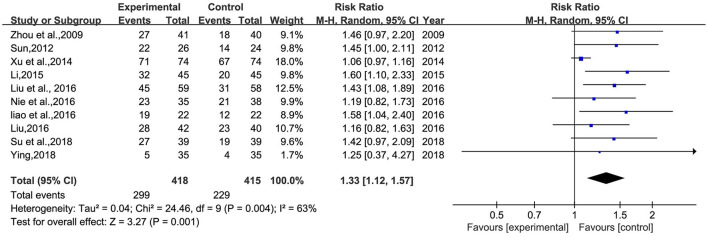
Meta-analysis of BHD and antidepressant drugs regarding total clinical effectiveness. Blue: Dichotomous.

**Figure 6:**
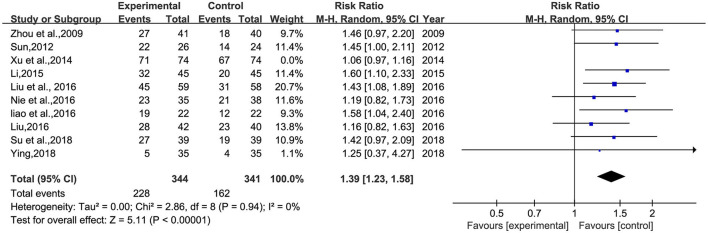
Meta-analysis of BHD and antidepressant drugs regarding total clinical effectiveness (9 items). Blue: Dichotomous.

A total of 10 studies (26–30, 32–34, 36, 38) involving 833 patients were included (Figure 5). Heterogeneity was tested (*p* = 0.001, *I*^2^ = 63%), and a meta-analysis was performed using a random-effects model. The results of the analysis showed that BHD was significantly better than that of the control group compared with conventional treatment alone, or with the addition of antidepressants. The difference was statistically significant [RR = 1.33, 95% CI (1.12, 1.57)]. The confidence interval of one (34) study in the figure differs significantly from the other studies, which was analyzed after a careful reading of the literature; this study was not grouped according to the randomized number table method and the duration of BHD continued for 3 months, much more than the other studies. A meta-analysis was performed after excluding this study, and the results showed RR = 1.39, 95% CI (1.23, 1.58) (Figure 6).

#### Neurological deficit score

**Figure 7:**
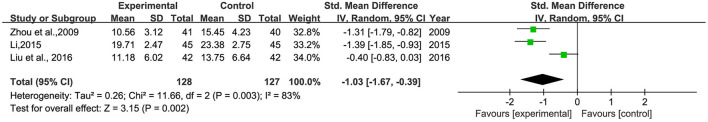
Meta-analysis of BHD and antidepressants regarding neurological deficit scores. Green: Continuous.

A total of three studies (26, 28, 38) involving 255 patients were included (Figure 7). Heterogeneity was tested (*p* = 0.002, *I*^2^ = 83%), and a meta-analysis was performed using a random-effects model. The analysis results showed that BHD could significantly improve the neurological deficit score in patients with PSD compared with conventional treatment alone or Western medicine. The difference was statistically significant [SMD = −1.03, 95% CI (−1.67, −0.39)]. The reason for the heterogeneity may be related to the different scoring criteria of different scales.

### Safety inspection

This study mainly evaluated the safety of BHD in treating PSD from the incidence of adverse reactions and the score of TESS.

#### The incidence of adverse reactions

**Figure 8:**
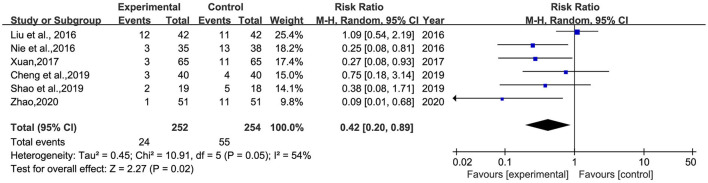
Meta-analysis of BHD and antidepressant drugs regarding the incidence of adverse reactions. Blue: Dichotomous.

Six studies (25, 28, 30, 31, 33, 37) reported the incidence of adverse reactions, involving 506 patients (Figure 8). Heterogeneity was tested (*p* = 0.02, *I*^2^ = 54%), and a meta-analysis was performed using a random-effects model. The results showed that the incidence of adverse reactions in the intervention group was significantly lower than that in the control group. The difference was statistically significant [RR = 0.42, 95% CI (0.20, 0.89)].

#### TESS score

**Figure 9:**
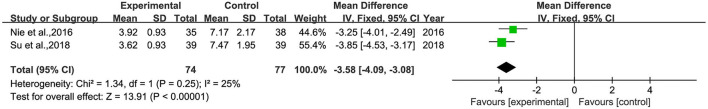
Meta-analysis of BHD and antidepressants regarding TESS scores. Green: Continuous.

Four studies (28, 30, 32, 36) reported the score of TESS, and two (30, 32) of them reported significant differences between the two groups, involving 151 patients (Figure 9). Heterogeneity was tested (*p* < 0.00001, *I*^2^ = 25%), and a meta-analysis was performed using a fixed-effects model. The analysis showed that the TESS scores of the intervention group were significantly lower than those of the control group, and the difference was statistically significant [MD = −3.58, 95% CI (−4.09, −3.08)].

Meanwhile, five studies (25, 28, 30, 31, 37) reported the occurrence of adverse effects after drug administration, such as headache, dizziness, insomnia, anxiety, drowsiness, dry mouth, nausea, diarrhea, gastrointestinal reactions, gray hair, and weight gain. Most of the symptoms were tolerated by patients or resolved on their own after discontinuation of the drug without affecting normal treatment. Only in one study (30) did the control patients withdraw from the study on their own due to severe gastrointestinal reactions. Most of the included studies were considered to include conventional treatment for stroke, and the medication regimens included multiple antihypertensive drugs, antiplatelet aggregation drugs, drugs to improve cerebral circulation, and so on. Their adverse drug reactions were complex and varied, and it is not certain whether adverse reactions occurred due to BHD.

### Sensitivity analysis

Sensitivity analysis was performed on the above outcome indicators. No significant changes in effect sizes and values occurred after excluding the included studies one by one. This conclusion indicates that the results of the meta-analysis are stable and credible.

### Publication risk assessment

**Figure 10:**
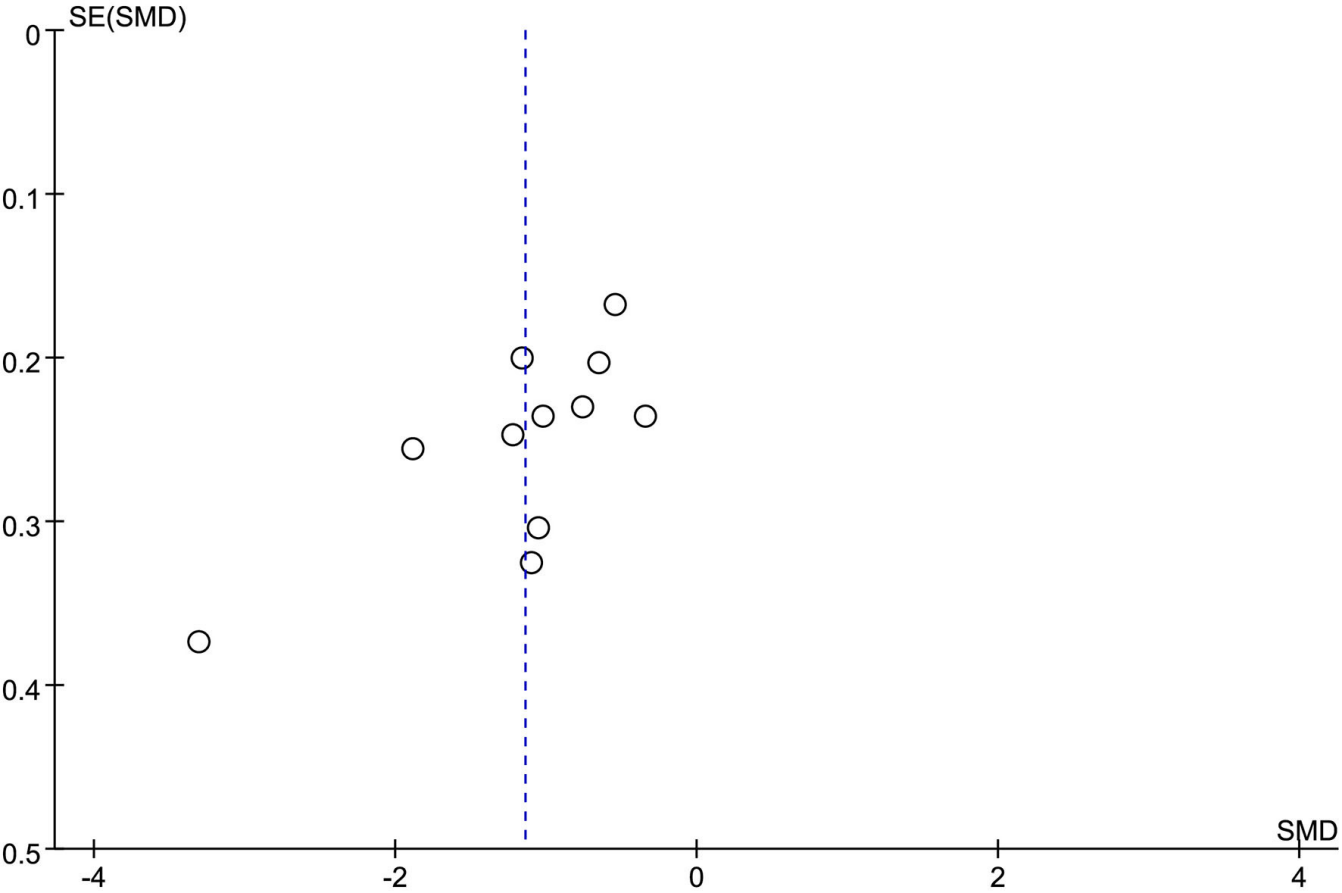
HAMD changes the value of published bias funnel plot.

**Figure 11:**
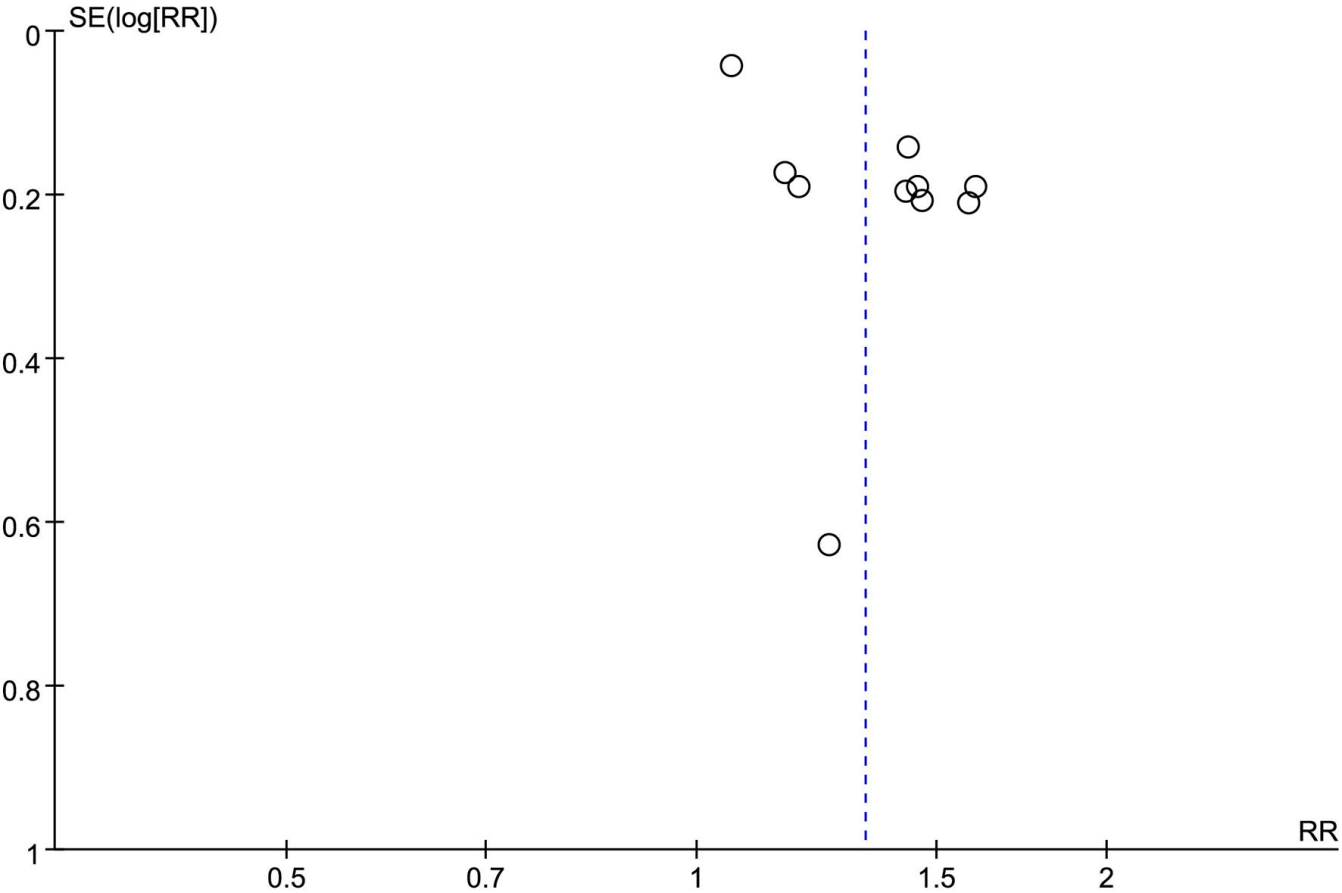
Efficient publication of biased funnel plots.

Publication bias analysis was performed using the funnel plot method for the change in the value of the outcome index, the changes of HAMD, and the total clinical effectiveness rate. The funnel plots were all asymmetrically distributed, suggesting the presence of publication bias. This may be related to the different HAMD versions, the small sample size, and the less rigorous implementation of the allocation concealment blinding method (Figures 10, 11).

## Discussion

This study is a meta-analysis of the effectiveness and safety of BHD for the treatment of PSD. A total of 15 studies involving 1,242 patients were included to compare the clinical efficacy of BHD with antidepressant drugs (fluoxetine, paroxetine hydrochloride, mirtazapine, and haloperidol melitrexin tablets) for the treatment of PSD. Meta-analysis showed that before and after treatment, the change of HAMD and the total clinical effective rate in the intervention group using BHD were higher than those in the control group, suggesting that BHD alone or in combination with antidepressant conventional drugs is likely to be superior to antidepressant drugs alone for the treatment of PSD. Meanwhile, subgroup analysis showed that BHD was administered for 4 weeks at a dose of 200 ml of herbal soup two times a day, and antidepressant efficacy appeared to be most pronounced. The neurological deficit score reflects the prognosis of stroke patients. The higher the patient's score, the worse the prognosis of stroke. Compared with the control group, the intervention group can significantly reduce the neurological deficit score of PSD patients, suggesting that BHD can significantly improve the prognosis of stroke patients. The safety of BHD was closely related to the TESS score and the incidence of adverse events. The TESS score was positively correlated with the severity of adverse symptoms and signs after taking the medication, and whether adverse reactions occur or not is the safety of taking the medicine directly. The results of the analysis showed that the TESS score and the incidence of adverse reactions in the intervention group were significantly lower than those in the control group. These results suggest that BHD alone or in combination with antidepressants for the treatment of PSD appears to have better clinical efficacy with no or fewer adverse effects and a higher safety profile.

PSD is one of the common complications of a stroke. About one-third of the survivors of stroke suffer from this depression, with a high incidence rate, which is an important factor hindering the recovery of neurological function and daily living ability of stroke patients. However, the clinical diagnosis and assessment of PSD are seriously inadequate, as the diagnosis relies on depression scale scores and clinical symptoms, which are subjective; and because stroke patients have aphasia or cognitive dysfunction, the condition cannot be effectively assessed, making PSD more difficult to diagnose and intervene. Meanwhile, modern medicine believes that the pathogenesis of PSD is complex and diverse, involving a variety of neurobiological dysfunctions under the influence of social and psychological factors, including neuroinflammatory responses, activation of the hypothalamic-pituitary-adrenal axis, neuronal cell plasticity, secondary degenerative changes, neurotransmitters, brain-derived neurotrophic factor transmission, and so on (39, 40). The complex pathogenesis of PSD has led to the lack of an objective evaluation method with recognized standard specificity. Blood and urine tests based on pathogenesis are used in combination or different combinations of the same type of indexes, and imaging methods are mostly focused on the prediction of PSD and fail to elaborate on its specificity with PSD (41). Thus, the current objective evaluation methods for PSD are confusing. Therefore, it causes much inconvenience to the basic and clinical research of PSD.

The goals of treatment for PSD are to alleviate depressive symptoms, improve quality of life, and reduce depression recurrence and stroke recurrence. However, the pathogenesis of PSD involves social, psychological, and physiological aspects, and there are limitations in treating PSD only from a single mechanism and target. Stroke survivors are more likely to be middle-aged, elderly, and frail, and in most cases even combined with multiple underlying diseases, and their adverse reactions to antidepressants are more pronounced. Second, in previous clinical studies, it was found that 10–30% of patients with major depressive disorder are resistant to antidepressants, unable to respond to antidepressants or only partially responding to antidepressants, and can still be accompanied by self-injury, suicide, depressive relapse, and other manifestations of the disease (42); in addition, some patients are resistant, fearful, and resistive to antidepressants; for many reasons, the clinical efficacy of traditional antidepressants in patients with PSD is not very satisfactory. Compared to conventional antidepressant drugs, BHD has many advantages in the context of evidence-based treatment, including multi-mechanism and multi-target therapeutic effects, high patient compliance, few adverse reactions in the treatment process, low drug side effects, and so on. It was confirmed that the occurrence of PSD was positively correlated with the degree of neurological deficits, while the occurrence of PSD further affected the recovery of neurological functions in stroke patients (43). The two interacted with each other and were mutually causal. Experiments have demonstrated that BHD can improve the content of various neurotransmitters in the brain, inhibit neural cell apoptosis, promote neural cell proliferation and differentiation, and exert neuroprotective effects from multiple genes, pathways, and targets (44–51). BHD, as a classical formula for tonifying Qi and invigorating Blood, is very suitable for stroke pathogenesis of Qi deficiency and Blood stasis, and has the effect of tonifying Qi and invigorating Blood, and resolving blood stasis and calming the mind. Clinical studies have also demonstrated that BHD improves depressive symptoms and promotes emotional recovery in patients with PSD.

Limitations in the current study. First, the quality of the included literature was generally low. Most of the literature failed to provide detailed information on their specific methodology, such as whether allocation concealment was performed and whether they were blinded. Although the baseline level of patients was balanced, there is still a high risk that randomization and blinding may be unclear. Second, the heterogeneity of included studies was high. Multiple factors may contribute to heterogeneity, such as the version of the HAMD score, the criteria for evaluating clinical effectiveness, the duration of the patient's illness, the degree of depression, the type and dose of the intervention medication, and the duration of administration. All of these factors may contribute to heterogeneity and influence the final treatment outcome. Finally, publication bias may have been present in this study. Although the two researchers ranked the literature strictly according to inclusion and exclusion criteria, no studies with negative findings were identified in this meta-analysis. This may be related to the fact that positive results are more likely to be published. In addition, although the two researchers conducted a comprehensive database search, we cannot exclude the possibility that some studies may have been missed.

## Conclusion

This systematic review and meta-analysis summarized in detail the efficacy of BHD in the treatment of PSD. Based on 15 RCTs, BHD was safe and effective in reducing HAMD scale scores, lowering neurological deficit scores, reducing the incidence of adverse effects, and improving treatment efficiency. However, due to the lack of methodological evidence, rigorously designed RCTs are still needed to support the clinical effectiveness of BHD for PSD.

## Data availability statement

The original contributions presented in the study are included in the article/supplementary material, further inquiries can be directed to the corresponding author/s.

## Author contributions

DG and GS participated in the research design. KZ and HS conducted a literature search and screened data extraction. KZ analyzed the data, did a statistical analysis, and wrote a manuscript. XL and WL participated in the correction of the manuscript. HJ and XZ made important contributions to the revision of this article. All authors reviewed the manuscript, read, and approved the final version of the manuscript.

## Funding

This article was supported by the second round of construction project of the National Academic School of Chinese Medicine Inheritance Studio of the State Administration of Traditional Chinese Medicine (No. 2019-28) and the Youth Project of Shandong Province Traditional Chinese Medicine Science and Technology Development Project (No. 2021Q115).

## Conflict of interest

The authors declare that the research was conducted in the absence of any commercial or financial relationships that could be construed as a potential conflict of interest.

## Publisher's note

All claims expressed in this article are solely those of the authors and do not necessarily represent those of their affiliated organizations, or those of the publisher, the editors and the reviewers. Any product that may be evaluated in this article, or claim that may be made by its manufacturer, is not guaranteed or endorsed by the publisher.

## Abbreviations

PSD: post stroke depression
HD: buying huanwu decoction
PRISMA: Preferred Reporting Items for Systematic Reviews and Meta-Analyses
RCT: Randomized controlled trial
CNKI: China National Knowledge Infrastructure
CBM: Chinese Biological Medicine
HAMD: the Hamilton Depression Scale
TESS: the Treatment Emergent Symptom Scale.
